# Gut microbial diversity in *Apis cerana indica* and *Apis florea* colonies: a comparative study

**DOI:** 10.3389/fvets.2023.1149876

**Published:** 2023-05-12

**Authors:** Khalid Ali Khan, D. N. Ganeshprasad, H. R. Sachin, Yogesh S. Shouche, Hamed A. Ghramh, A. H. Sneharani

**Affiliations:** ^1^Applied College, Mahala Campus, King Khalid University, Abha, Saudi Arabia; ^2^Unit of Bee Research and Honey Production, King Khalid University, Abha, Saudi Arabia; ^3^Research Center for Advanced Materials Science (RCAMS), King Khalid University, Abha, Saudi Arabia; ^4^Department of Studies and Research in Biochemistry, Jnana Kaveri Post Graduate Centre, Mangalore University, Chikka Aluvara, Karnataka, India; ^5^National Centre for Microbial Resource, National Centre for Cell Science, Pune, India

**Keywords:** dwarf honey bee, *Apis cerana indica*, *Apis florea*, gut microbiota, pollinator

## Abstract

**Introduction:**

Honey bee gut microbiota have an important role in host health, nutrition, host-symbiont interaction, and interaction behavior with the surrounding environment. Recent discoveries of strain-level variation, characteristics of protective and nutritional capabilities, and reports of eco-physiological significance to the microbial community have emphasized the importance of honey bee gut microbiota. Many regions of Asia and Africa are inhabited by the dwarf honey bee, *Apis florea*. Studying its microflora and potential for pollination is therefore of foremost importance.

**Methods:**

In the present investigation, we aimed to explore the gut bacteriobiome composition of two distinct honey bee species, *Apis florea* and *Apis cerana indica* using high throughput sequencing. Functional predictions of *bee* gut bacterial communities using PICRUSt2 was carried out.

**Results and discussion:**

The phylum Proteobacteria dominated the bacterial community in both *A. cerana indica* (50.1%) and *A. florea* (86.7%), followed by Firmicutes (26.29 and 12.81%), Bacteroidetes (23.19 and 0.04%) and Actinobacteria (0.4 and 0.02%) respectively. The gut bacteria of *A. cerana indica* was more diverse than that of *A. florea*. The observed variations in bacterial genomic diversity among these critical pollinator species may have been influenced by the apiary management techniques, ecological adaptation factors or habitat size. These variations can have a significant effect in understanding host-symbiont interactions and functioning of gut microbiota highlighting the importance of metagenomic survey in understanding microbial community ecology and evolution. This is the first comparative study on variation in bacterial diversity between two Asian honey bees.

## 1. Introduction

Honey bees are key pollinators of many crops for human use and provide valuable commodities such as honey and wax, making the gut microbiota of honey bees an interesting community to explore (1). Gut bacteria accomplish a wide range of functions that are vital to the host’s metabolism and overall health. Many living organisms benefit from gut microbial populations, from digesting indigestible polysaccharides to immunomodulation (1). Honey bees and their bumble bee relatives are globally important as they pollinate a vast majority of wild flowering plants and crops in the world. Given these aspects, the gut microbiota of social bees offers a promising new paradigm for studies on genetic diversity and the evolution of host-associated bacterial communities. The rapid decline in the bee populations has set a focus on possible factors that affect their health, especially their microbiota (2–4). There are significant functional differences in the gut bacterial communities of relative host species of honey bees. The gut microbiota of honey bees is becoming a prominent model for investigating genetic variation as well as the features that have led to the evolution of bacterial populations that are associated with hosts.

The microbiota is most common in the distal gastrointestinal system, where it assists in the breakdown and degradation of plant cell wall components (5). Gut bacteria are primarily transferred through social interactions in both bees and primates (6). Many invertebrate gut communities, on the other hand, have unpredictable compositions dominated by bacteria from environmental sources (7, 8). The reared species of honey bee have occupied a dominant position in commercial pollination around the world, as these are highly social bees. Yet, on the other hand, wild bees are useful pollinators, too. It is an efficient pollinator of numerous crops worldwide. Because of the honey bee’s significance to the agro-ecosystems in its native habitat, its conservation is highly anticipated (9). Due to limited understanding of their behavioral mechanism for nesting, perhaps their contribution toward pollination is underestimated (10). Modern apiculture, however, today suffers from several challenges, including parasitic mites, honey bee diseases, the inability of honey bees to function at low temperatures and adverse weather conditions. The general utility of honey bees as agricultural pollinators is confronted by these problems (10). This adds to the concern of beekeepers, insect-pollinated crop breeders and policy officials over fast growing reductions in honey bee populations (Colony Collapse Disorder) (11). Dwarf honeybees potential role in natural and agricultural systems offers a multidisciplinary perspective on various aspects. Production of lesser honey is the main limitation of *A. florea*, and hence not much attention has been given. However, the emendable pollination efficiency of this bee has considerably contributed to the varied flora in the tropical region, when considering how pollinator loss affects ecosystem stability and food security worldwide, the role of the dwarf honeybee *Apis florea* acquires vital significance (12).

The bacterial communities of wild flies are more diverse and vary in comparison to domesticated species (13), involving honey bee gut bacteria (14). Significant differences in microbiota diversity observed in these essential pollinator species could be associated with changes in bee management strategies, habitat size, or differential ecological evolution (15). Given the importance of metagenomic efforts to study the ecology and evolution of microbial populations, such alterations are expected to have an impact on gut flora interactions between hosts and symbionts. Despite advances in understanding of host-associated bacterial community diversity and its functional consequences, little is known about host species diversity variations or the underlying mechanisms that regulate and maintain diversification within or among hosts (15).

Among the few studies that have explained the bacterial community associated with the dwarf honey bee are as follows; an observation on lactic acid bacteria (LAB) in the crop of *A. florea*, assessment of bacterial communities in *A. florea* larvae (16, 17), and diversification of gut bacteria of a closely related species, *Apis andreniformis* (18).

Using targeted 16S rRNA gene amplicon sequencing, bacterial community composition profile of *A. cerana indica* and *A. florea* from the Indian subcontinent was studied. The microbial communities of *A. florea*, a pollinator, were compared with *A. cerana indica* with emphasis on ecosystem stabilization and commercial purposes, and we aimed to study the significant difference between the gut microbial compositions as well as major differences in functions.

## 2. Methodology

### 2.1. Collection of samples and bee gut dissection

A total of 60 worker honey bees (30 of each species) of *A. cerana indica* (four bee colonies from an apiary) and from three *A. florea* colonies were obtained in the Western Ghats of southern India’s forest region (Lat:12.14455 DMS N 12° 8′ 40.38″, Long: 75.9382 DMS E 75° 56′ 17.519″, College of Forestry, Ponnampet, Kodagu, India and Lat:12.55821 DMS N 12° 33′ 29.556″, Long:75.95338 DMS E 75° 57′ 12.167″, Mavina Halla Forest, Kodagu, Karnataka, India respectively). The sampling sites were located far among the bee colonies. The method used for sampling was followed as per the available literature (15, 19). During the same day, live honey bees were transferred in small cages to the laboratory for dissection; all honey bees were cold anaesthetized and were surface-sterilized in sterile falcon tubes, with sodium hypochlorite (7%) and ethanol (70%), then washed four times using sterile phosphate-buffered saline (PBS) (20). The complete alimentary canals of honey bees were dissected under aseptic conditions using saline (0.9%) after the stinger was removed with sterilized forceps. The dissected whole guts were transferred directly to phosphate-buffered saline (PBS) and preserved at −20°C for further analysis.

### 2.2. Community DNA extraction

In two separate tubes, the alimentary tracts of worker honey bees (*n* = 60, 30 for each species of *A. cerana indica* and *A. florea*) were collected in 1 mL PBS. Genomic DNA was extracted using Qiagen Tissue kit (Genomic DNA extraction kit, United States) and the subsequent steps were carried out according to the manufacturer’s instructions. As detailed by (21), the concentration of DNA was determined using a NanoDropND-1000 spectrophotometer (Willington, United States) and the quality was determined using a 0.8% agarose gel. The DNA was kept at −20°C until further processing.

### 2.3. High throughput sequencing

To study the bacterial diversity in honey bee guts, NGS libraries were constructed by targeting V4 region of 16S rRNA gene with primers 515F (5′-GTGCCAGCMGCCGGTAA-3′) and 806R (5′-GGACTACHVGGGTWTCTAAT-3′) (21). The library construction of 16S rRNA gene amplicons was carried out in accordance with Illumina protocols (United States). Purified libraries were subsequently pooled in equimolar concentrations and sequenced on the Illumina MiSeq system using 2 × 250 bp v2 Chemistry.

### 2.4. Bioinformatics study

The raw reads obtained from high-throughput sequence analysis were assembled using the FLASH tool (22). The pre-processing of raw Fastq files were carried out using MOTHUR v1.32 to remove adaptors, low quality and ambiguous reads which minimizes the occurrence of erroneous sequences to the dataset. The reference-based OTU picking method was used to select operational taxonomic units (OTUs) against SILVA (v132) (23). QIIME v1.8 was used to calculate α-biodiversity indices such as Chao1, ACE and Shannon (diversity), once the sequencing depth has been set for all samples (24). Furthermore, PICRUSt2 tool was used to perform imputed metagenomics for the functional analysis of bacterial communities (25). The obtained dataset was analyzed using the KEGG (Kyoto Encyclopedia of Genes and Genomes) database (26).

## 3. Results

### 3.1. Characterization of microbial diversity

**Figure 1:**
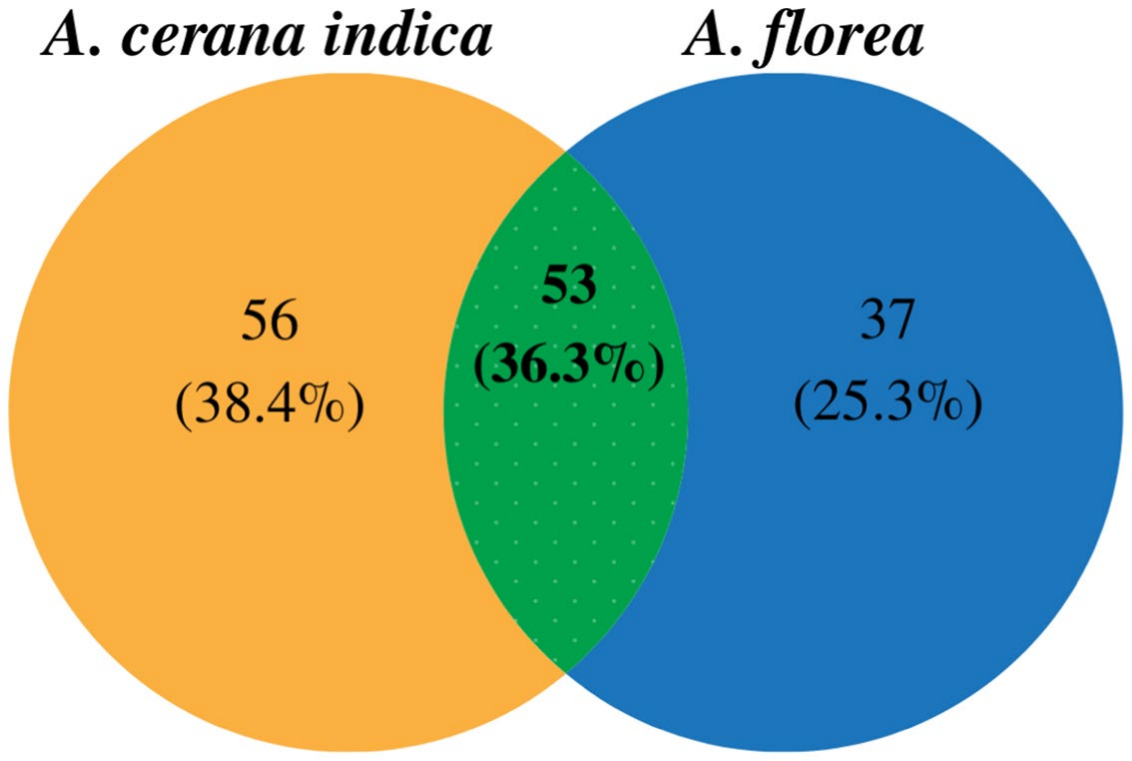
Venn diagram indicating unique and shared OTUs at genus level among *A. cerana indica* and *A. florea* of Asian honey bees.

High-throughput sequencing and the quality editing of the 16S rRNA gene in *A. florea* and *A. cerana indica* yielded 0.29 million quality reads, those were used for further data analysis. Sequences were taxonomically assigned using the reference database, yielding 867 operational taxonomic units (OTUs). For 16S rRNA amplicon sequencing, the mean Good’s coverage of *A. florea* and *A. cerana indica* samples was 82.81 ± 17.19% (mean ± SD), indicating that the majority of diversity was captured. When assessing bacterial diversity, the alpha diversity assessment of *A. florea* and *A. cerana indica* guts by means of species richness and the non-parametric estimation of shannon index indicated significant differences between them (Table 1). The bacterial species richness of *A. florea* (850.84) worker bees was found to be higher than that of *A. cerana indica* (498.60). The non-parametric estimation of shannon index for bacterial communities also differed between *A. florea* gut (3.12) and *A. cerana indica* gut (3.29). This finding suggests that the gut of *A. cerana indica* has more bacterial diversity than *A. florea*. This observation showed that both the species shared 36.3% OTUs at the genus level (Figure 1).

**Table 1:** Alpha diversity estimation.

	Chao1	Observed OTUs	Shannon	ACE
Gut bacteria of *A. florea*	850.84	589	3.12	858.48
Gut bacteria of *A. cerana indica*	498.60	278	3.29	514.46

Non-parametric alpha diversity was calculated for *A. florea* and *A. cerana indica* gut bacteria.

### 3.2. Bacterial community profiles in the guts of *Apis florea* and *Apis cerana indica*

**Figure 2:**
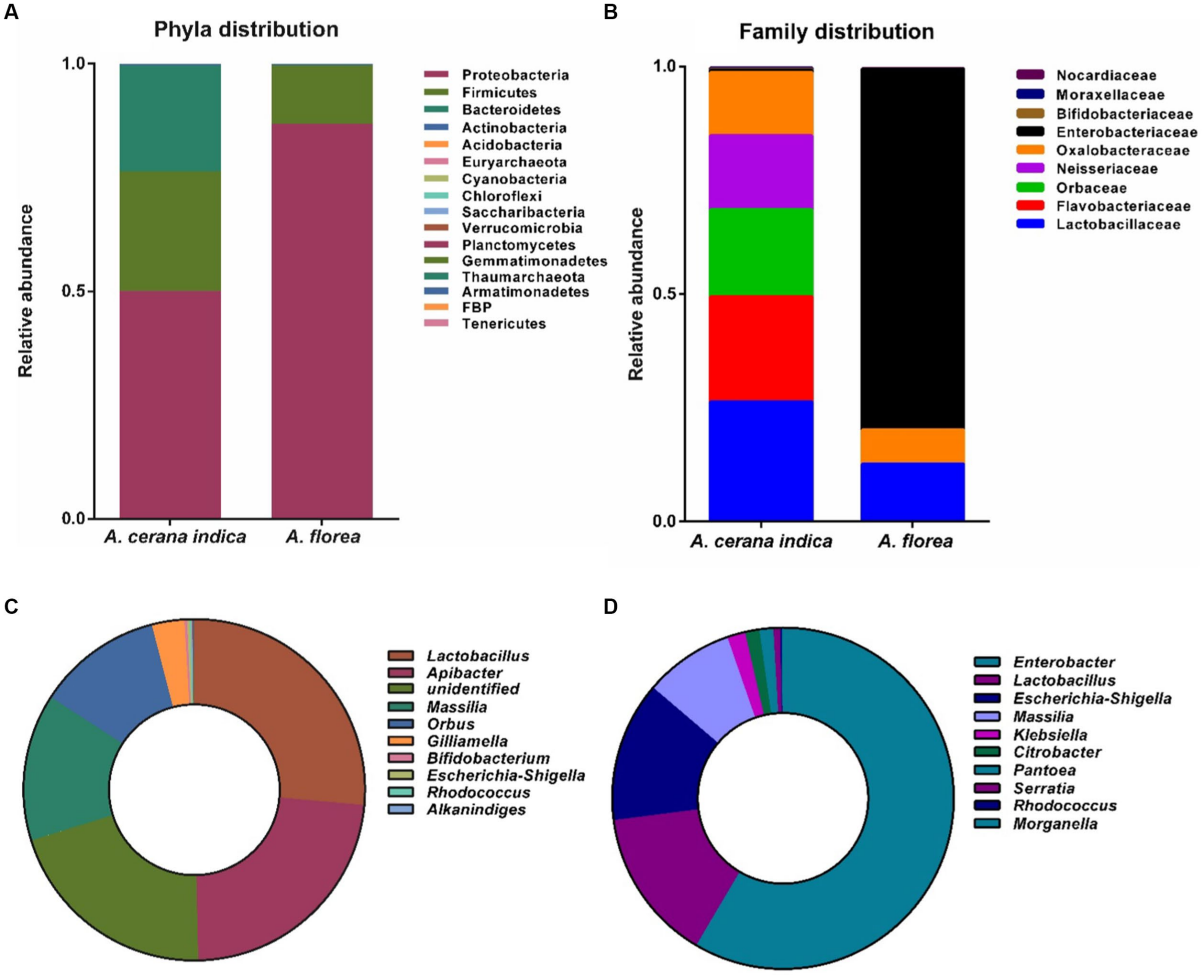
Bacterial taxa distributions of phylum, family and genus at different phylogenetic levels in the gut of *A. cerana indica* and *A. florea*. **(A)** Bacterial phyla. **(B)** Bacterial family. **(C)** Bacterial genera in the gut of *A. cerana indica*. **(D)** Bacterial genera in the gut of *A. florea.*

Amplicon sequencing of the 16S rRNA gene detected 16 and 12 bacterial phyla in the guts of *A. cerana indica* and *A. florea*, respectively (Figure 2A). In *A. cerana indica* gut bacterial phyla distributions were Proteobacteria (50.09%), Firmicutes (26.29%), Bacteroidetes (23.19%), Actinobacteria (0.4%) and the remaining (0.03%) comprised the minor phyla. Acidobacteria, Euryarchaeota, Cyanobacteria, Chloroflexi, Saccharibacteria, Verrucomicrobia, Planctomycetes, Gemmatimonadetes, Thaumarchaeota, Armatimonadetes, FBP and Tenericutes. The following bacterial phyla were found in the gut of *A. florea*: Proteobacteria and Firmicutes (86.86 and 12.81% respectively), Actinobacteria (0.27%), Bacteroidetes (0.04%) were the predominant phyla, and the rest (0.02%). Acidobacteria, Verrucomicrobia, Cyanobacteria, Nitrospirae, Chloroflexi, Planctomycetes, Tectomicrobia, and Lentisphaerae are the minor phyla. The proportion of composition was not reported in Figure 2A because the abundance of 12 minor phyla was quite less. The phylum of bacteria Proteobacteria outnumbered all other bacterial phyla in the whole gut of honey bee, *A. florea*, whereas Proteobacteria, Firmicutes and Bacteroidetes were found to be the major phyla in the gut of *A. cerana indica*.

OTUs with ≥0.1% abundance that differed significantly between *A. cerana indica* and *A. florea* were filtered at the family level (Figure 2B). The family-level distributions of bacteria in the whole gut of *A. cerana indica* were Lactobacillaceae (26.39%), Flavobacteriaceae (23.21%), Orbaceae (19.12%), Neisseriaceae (16.3%), Oxalobacteraceae (13.91%), Enterobacteriaceae (0.53%), Bifidobacteriaceae (0.27%), Moraxellaceae (0.15%) and Nocardiaceae (0.12%). In the whole gut of honey bee, *A. florea*, the bacterial families were classified as Enterobacteriaceae (79.47%) and Lactobacillaceae (12.75%) as dominant families; Oxalobacteraceae (7.45%) as next dominant family, and the minor families were Nocardiaceae, Micrococcaceae, Bifidobacteriaceae, Staphylococcaceae, and Prevotellaceae. The bacterial family’s composition percentage, which was <1%, was not included in Figure 2B.

At genera level, OTUs with ≥0.1% abundance were filtered (Figures 2C,D) and distributions of the bacterial genera in the whole gut of *A. cerana indica* were *Lactobacillus* (26.41%), *Apibacter* (23.22%), unidentified bacteria (20.58%), *Massilia* (13.92%), *Orbus* (11.86%), *Gilliamella* (3.08%), *Bifidobacterium* (0.26%), *Escherichia-Shigella* (0.24%), *Rhodococcus* (0.12%) and *Alkanindiges* (0.11%) (Figure 2C). Genera in *A. florea* gut were *Enterobacter* and *Lactobacillus* (58.49 and 14.47% respectively) as predominant taxa*, Escherichia-Shigella* (13.29%), *Massilia* (8.45%) and *Klebsiella* (1.71%) as major genera; *Citrobacter, Pantoea, Serratia, Rhodococcus* and *Morganella* genera are less prevalent (Figure 2D). The bacterial genera composition percentage, which was <1%, was not shown in Figures 2C,D.

### 3.3. Imputed metagenomic functions of bacterial community

**Figure 3:**
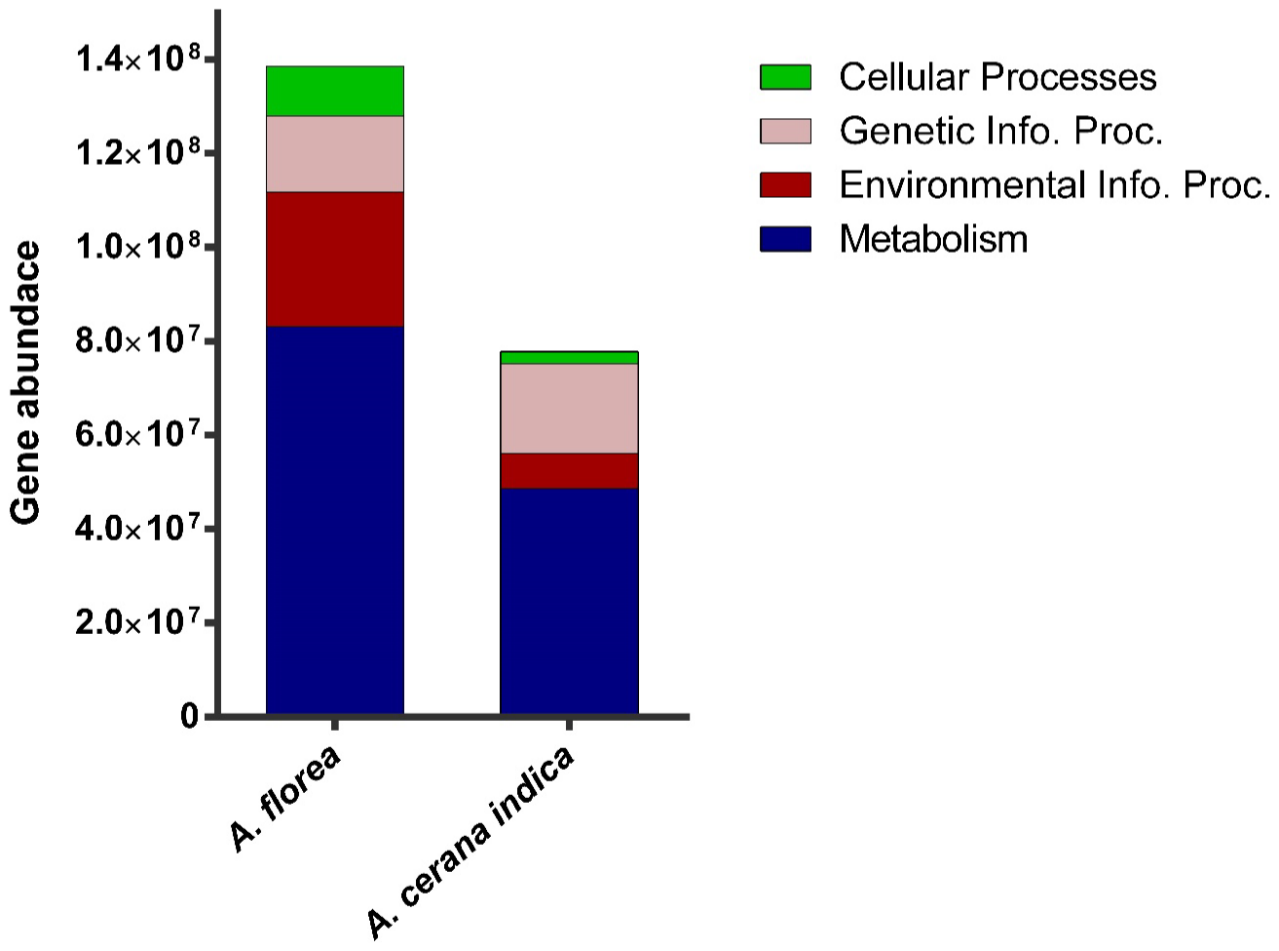
Metagenomic imputation. The distribution of metabolism, environmental information processing (Environmental Info. Proc.), genetic information processing (Genetic Info. Proc.) and cellular processes-related gene families in the gut bacterial taxa of *A. florea* and *A. cerana indica*.

Functional predictions of *A. florea* and *A. cerana indica* gut bacterial communities using PICRUSt2 revealed higher metabolism-related gene families, inferring that these bacterial populations play a significant role in the breakdown of complex macromolecules. Additionally, gene families involved in environmental info. proc. (Information processing), genetic info. proc. related and cellular processes were identified (Figure 3).

**Figure 4:**
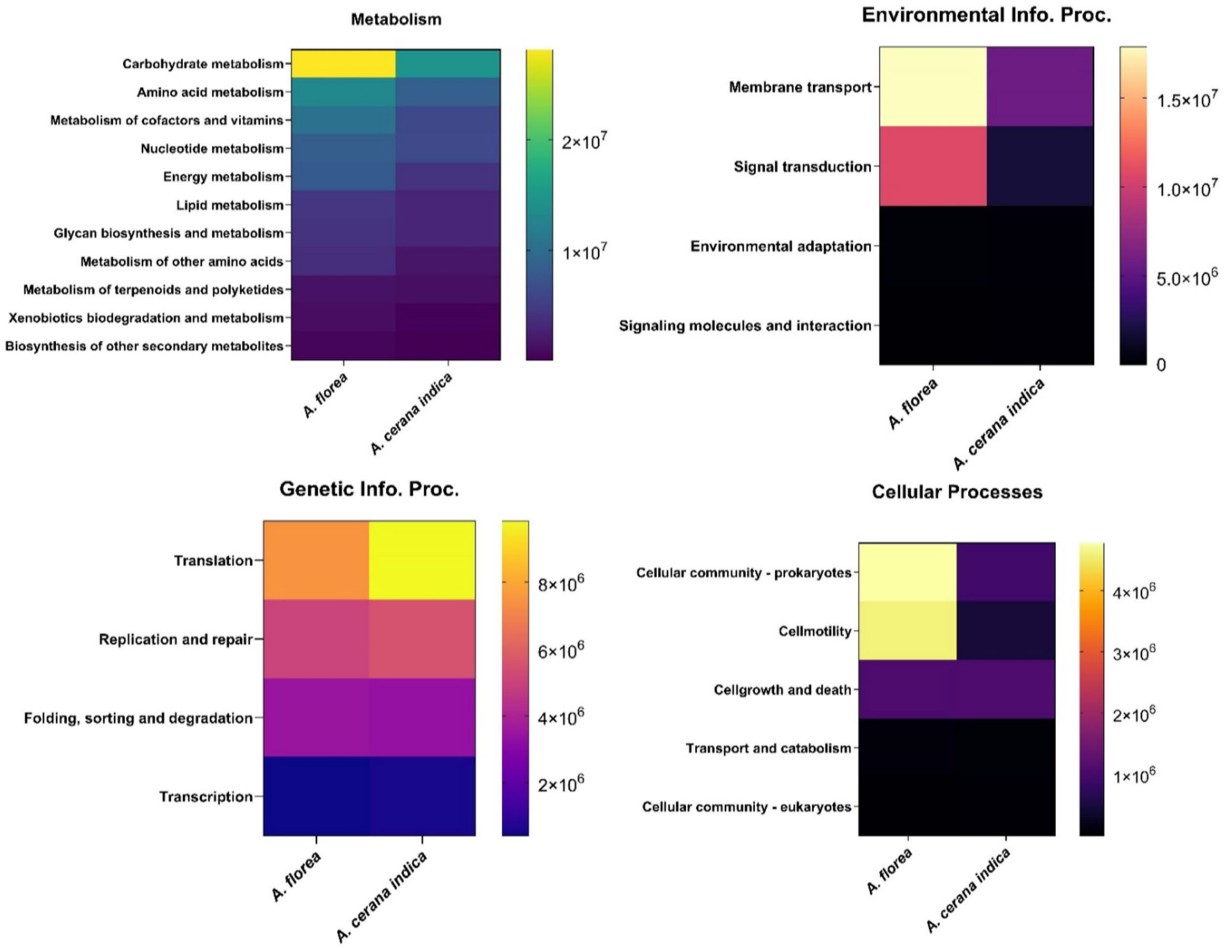
Heat map detailing the functional imputations. It represents the differential abundance of gene families among the gut bacterial communities of *A. florea* and *A. cerana indica*.

Assortment of each functional module such as metabolism is correspondingly prevalent in carbohydrate metabolism (32.4%) and amino acid metabolism (16.43%) respectively: Vitamin and cofactor metabolism (12.77%), nucleotide metabolism (11.13%), energy metabolism (9.1%), lipid metabolism (5.6%), glycan biosynthesis and metabolism (5.35%) and metabolism of other amino acids (4.05%). Similarly, Membrane Transport (64.65%), Signal transduction (34.42%); Translation (48.53%), Replication and repair (29.69%); Cellular motility (39.66%) were identified as significant contributors to environmental info. proc. and genetic info. proc. and cellular functions, respectively (Figure 4).

## 4. Discussion

Our study highlights the gut bacterial diversity of two different species of honey bees of India using a high throughput approach. There were quantitative differences in the prevalence of bacterial taxa among *A. cerana indica* and *A. florea*.

*Lactobacillus*, *Apibacter*, *Massilia*, *Orbus*, *Gilliamella*, *Bifidobacterium*, *Escherichia-Shigella*, *Rhodococcus* and *Alkanindiges* were the predominant bacterial genera observed in the gut of *A. cerana indica*. *Lactobacillus* and *Bifidobacterium*, which are often present in honey bee guts, have antibacterial properties and are involved in the fermentation of carbohydrates (27). The genus *Apibacter* occur as host-specific members of the bee gut microbiota and various *Apibacter* species or strains can be found in different bee hosts (27).

In the gut of *A. mellifera*, *Snodgrassella alvi* as well as *Gilliamella apicola* are abundant (28). In our analysis, *G. apicola* was found abundant whereas *S. alvi* were found in traces. *G. apicola* can degrade pectin and other cell wall components of pollen grains (29).

*Enterobacter*, *Lactobacillus*, *Klebsiella*, *Escherichia-Shigella*, *Citrobacter*, *Massilia*, *Pantoea*, *Serratia*, *Morganella* and *Rhodococcus*, are the most common taxa discovered in the guts of *A. florea* worker bees. Because of anaerobic and haloalkaliphilic environments, *Prevotella* and *Massilia* cannot be cultivated in standard laboratory settings. *Prevotella, Rhodococcus*, *Massilia,* and *Morganella* have not been reported in gut microbiota of honey bee in the literature. However, *Massilia* and *Prevotella* are gut mutualists of Red Mason Bee, *Osmia bicornis* and bark beetle (*Dendroctonus rhizophagus*) (30, 31) respectively, known to interact with arthropods providing fitness benefits and are the most common gut bacteria.

Firmicutes and Proteobacteria were discovered in *A. florea* in our investigation, which was consistent with previous studies in *A. florea* and *A. cerana indica* (17, 32). In the present study, *Enterobacter* occur predominantly in *A. florea* gut followed by the genus *Klebsiella*. In comparison to *A. cerana indica*, *A. florea* encountered higher environmental bacterial taxa, opportunistic in nature, such as *Klebsiella* and *Enterobacter* species. Because they are wild pollinators and cannot be domesticated, the presence of non-core gut microbes in *A. florea* is linked to exposure to a diverse habitat and floral diversity (33). Production of protease and lipase enzymes have been found in *Klebsiella* and *Enterobacter* genera (34–36). The gut associated *Klebsiella* sp. found to produce multiple polysaccharide degrading enzymes in apple snail and wild dwarf bee *A. florea* (37, 38). In the gut of honey bees, these bacteria may aid in the digestion of pollen grains (32). On the other hand, the gut of *A. cerana indica* found to share bacterial species with other *Apis* clade that were already reported in the literature.

During nectar and pollen gathering, insects acquire and discharge microorganisms on floral surfaces, shaping the flower microbiome. These microbes found in flowers are mostly bacteria. According to a recent study, these floral occupants can act as facilitators in plant-pollinator interaction (39). Insect pollination is a natural process that involves the transfer of bacteria between flowers to seeds. Varieties of bacterial taxa that may be transferred by insects have been found by previous community-profiling techniques used on the seed microbiome of diverse plant species. *Enterobacteriaceae* spp. are present both in seeds, flowers and insect visitors (*A. mellifera*), indicating a potential microbial transmission from insect pollinators through the floral route to the seed. Insect pollinators may play a role in determining the composition of the seed microbiome which is yet to be understood (39). Cultivated bacteria belonged to the phyla Proteobacteria, Actinobacteria, and Firmicutes, with notable species differences amongst pollen species. Proteobacteria was shown to be the predominant phylum in almost pollen species, followed by Actinobacteria, Firmicutes and Acidobacteria, according to next generation sequencing. In our study, also we observed the maximal presence of Proteobacteria, Firmicutes and Actinobacteria which are found in the other pollinator species (40).

The structure and diversity of pollen microbiota were significantly impacted by plant species and pollination type. Insect-pollinated species had more similar microbiome than wind-pollinated species, implying a leveling impact by insect vectors (40). *Acinetobacter* spp. stimulates pollen germination and induces growth density along with releasing proteins demonstrating that germination stimulation improves bacterial fitness. *Acinetobacter* directly induces and benefits from pollen germination and bursting. Further investigation into microbe-pollen interactions could help to improve several elements of pollination ecology, such as floral microbial ecology, pollinator nutrition absorption from pollen, and pollen germination cues for plant reproduction (41).

Similarly, gut microbial communities were found to fall into two separate compositional categories in a 16S rRNA investigation of 28 bumble bee species in China, with varying frequencies between species (42). The first type was the common corbiculate bee-specific microbial community, which included *S. alvi*, *G. apicola*, *Lactobacillus* Firm4 and Lactobacillus Firm5. The second type was dominated by Enterobacteriaceae and other Proteobacteria, as well as *Lactobacillus* sp. found in the environment (14). Overall, it signifies the influence of gut microbiota on bee health and provides a good base for the study model to analyze evolutionary pattern of gut symbionts.

The shift to non-core bacterial species occurs in wild bees, according to in-depth approaches using experimental colonies of the *Bombus terrestris*, a European bumble bee, as they are exposed to stress and protruding colonizers from environmental sources, and they are more likely to encounter non-core Enterobacteriaceae in their guts than bees from indoor colonies (43–45). These findings indicate that young adults acquire the characteristic gut symbionts, resulting in a rather stable ‘normal’ microbial community of co-adapted bacteria, but that individual bee microbial communities can be conquered by opportunistic bacteria over time. The cause of these aggravations is unknown (14).

The findings of this study prompted an interest in learning more about habitat functional biodiversity. The functional diversity of bacterial gene pool in whole guts of *A. cerana indica* and *A. florea* was determined using the PICRUSt2 tool. The results revealed a diverse range of genetic variation involved in numerous essential processes *viz.*, environmental information: signaling, signal transduction, membrane transport related gene families; genetic information: replication and repair, transcription and translation; cellular processes: cell growth and death, cellular motility, transport and catabolism, and carbohydrate, protein, and other biomolecule metabolism, comprising the production of some secondary metabolites. In the bacterial communities of *A. cerana indica* and *A. florea*, we discovered a ~61% greater congregation of gene families with metabolic functions.

## 5. Conclusion

The gut bacterial taxa of *A. cerana indica* are more diversified than those of *A. florea*. The high bacterial diversification in the whole gut microbiota of *A. cerana indica* may provide greater metabolic flexibility, allowing feeding on a wider range of pollen sources and consequently adaptation to varying environmental circumstances more rapidly. It is also feasible that a gut microbiota with less diversity but better adaptation to local conditions will be more advantageous than one with higher variety. These assumptions can be evaluated in honey bees, revealing new information about the function of bacterial diversity in host-associated bacterial populations and honey bee health. Dwarf bee *Apis florea* is an excellent pollinator of many important crops. The gut bacterial diversity analysis revealed that *Apis florea* comprises more bacteria taxa that inhabit pollination environment. Hence, *Apis florea* is greater pollinator compared to *Apis cerana indica*. It is important to assess *A. florea*’s potential for pollinating the native vegetation and for maintaining plant biodiversity in both forests and arid environments. It is also necessary to investigate the possibilities for making use of the bee’s capability for pollination, honey production, and improved agricultural economy. The value of *A. florea* as a pollinator considerably outweighs the value of the honey; hence, it should be allowed to persist in its natural habitats. Understanding the basic properties of symbiotic gut microbes has the potential to reveal previously unknown mechanisms and assist in the identification of pollinator species and their potential role in pollination.

## Data availability statement

The datasets presented in this study can be found in online repositories. The names of the repository/repositories and accession number(s) can be found in the article/Supplementary material.

## Author contributions

DG conducted the research, performed the data analyses, and wrote and reviewed the manuscript. HS, KK, and HG edited and reviewed the manuscript. YS provided experimental support. AS planned, supervised, organized the experiment, and wrote and reviewed the manuscript. All authors contributed to the article and approved the submitted version.

## Conflict of interest

The authors declare that the research was conducted in the absence of any commercial or financial relationships that could be construed as a potential conflict of interest.

## Publisher’s note

All claims expressed in this article are solely those of the authors and do not necessarily represent those of their affiliated organizations, or those of the publisher, the editors and the reviewers. Any product that may be evaluated in this article, or claim that may be made by its manufacturer, is not guaranteed or endorsed by the publisher.
